# Expression of immune checkpoint molecules on adult and neonatal T-cells

**DOI:** 10.1007/s12026-022-09340-6

**Published:** 2022-11-23

**Authors:** Stefanie Dietz, Kriszta Molnar, Hannah Riedel, Laura Haag, Bärbel Spring, Thorsten W. Orlikowsky, Christian F. Poets, Christian Gille, Natascha Köstlin-Gille

**Affiliations:** 1 Department of Neonatology, Tübingen University Children’s Hospital, Tübingen, Germany; 2 Department of Neonatology, Heidelberg University Children´S Hospital, Heidelberg, Germany; 3 Section of Neonatology, Aachen University Children´S Hospital, Aachen, Germany

**Keywords:** T-cell proliferation, Immune-checkpoint molecules, Co-stimulatory molecules, Neonatal sepsis

## Abstract

Term and especially preterm neonates are much more susceptible to serious bacterial infections than adults. But not only the susceptibility to infection is increased in neonates, but also their risk for developing post-inflammatory diseases such as bronchopulmonary dysplasia (BPD) and periventricular leukomalacia (PVL). This may be due to an impaired ability to terminate inflammation. In the study presented here, we aimed to investigate the proliferative response and the expression of immune-checkpoint molecules (ICM) and activation markers on neonatal T-cells in comparison to adult T-cells with the hypothesis that an increased activation of neonatal T-cells may contribute to the failure of inflammation resolution observed in neonates. We show that neonatal CD4^+^ and CD8^+^ T-cells show an increased proliferative capacity and an increased expression of activation markers compared to adult T-cells upon stimulation with OKT3 as well as a decreased expression of ICM, especially PD-L1 on their surface. This decreased expression of PD-L1 by neonatal T-cells was also observed after stimulation with GBS, but not after stimulation with *E. coli*, the two most important pathogens in neonatal sepsis. Expression of the T-cell receptor CD3 and the co-stimulatory molecule CD28 did not differ between adult and neonatal T-cells upon bacterial stimulation. Decreased expression of ICM upon T-cell activation may be a reason for the increased risk of neonates to develop post-inflammatory diseases.

**Supplementary Information:**

The online version contains supplementary material available at 10.1007/s12026-022-09340-6.

## Introduction

Neonatal sepsis is one of the leading causes of infant death and, despite advances in the neonatal care, often results in poor outcome among survivors [1–3]. The incidence of neonatal sepsis in term neonates (≥ 37 weeks’ of gestation) is about 0.05%, but it can increase to up to 40% in very low birth weight infants (VLBWI) [4, 5].‍ According to a WHO estimate, the incidence of sepsis is decreasing; however, still 10% of neonatal deaths are caused by sepsis or pneumonia globally [6, 7].

Compared to adults, term and especially preterm neonates are much more vulnerable to serious bacterial infections [8, 9]. Interestingly, not only their susceptibility to infection is elevated, but also the termination of inflammation seems to be disturbed [10], leading to dysregulated and prolonged inflammation. This failure of inflammation resolution can cause organ damage and may contribute to the development of post-inflammatory diseases such as bronchopulmonary dysplasia (BPD) and periventricular leukomalacia (PVL), thereby significantly influencing long-term outcome [11, 12].

In adults, the host immune response to sepsis is traditionally considered to be characterized by an initial hyper-inflammatory phase, primarily driven by innate immune cells and a protracted anti-inflammatory phase leading to persistent immunosuppression and recurrent infections [13]. This second phase of sepsis was shown to include a dysfunctional adaptive immune response [14] with T-cell exhaustion playing a major role. T-cell exhaustion is a progressive loss of T-cell functions in the presence of high antigen load. Exhausted T-cells express high levels of immune checkpoint molecules (ICM) like programmed cell death protein 1 (PD-1) and show decreased proliferative capacity [15].

ICM are inhibitory receptors expressed by immune cells and mediating immunosuppressive signaling pathways in the context of T-cell activation. ICM play an important role in tolerance to auto-antigens and in the regulation of inflammation responses. In addition, certain pathogens, tumors, and leukemias use the expression of ICM to evade the T-cell response (immune escape) [16–18]. In adult sepsis, increased expression of ICM, especially of expression of the PD-1/programmed death ligand 1 (PD-L1) axis, was correlated with the risk to develop nosocomial infections [19] and blockage of the PD-1/PD-L1 axis resulted in increased survival in experimental sepsis [20] [21].

The immunosuppression induced by sepsis has not yet been defined in neonates [22]. Actually, term and especially preterm neonates show a decreased capacity for terminating inflammation, a phenomenon called sustained inflammation [10, 11, 23, 24].

Here, we aimed to investigate the proliferative response and the activation state, as well as the expression of ICM on neonatal T-cells in comparison to adult T-cells with the hypothesis that decreased upregulation of ICM on neonatal T-cells upon bacterial stimulation may contribute to sustained inflammation. We show that upon stimulation with OKT3 neonatal CD4^+^ and CD8^+^ T-cells exhibit (1) an increased proliferative capacity and increased expression of activation markers in comparison to adult T-cells and (2) a decreased expression of ICM, especially PD-L1 on their surface. (3) The decreased expression of ICM by neonatal CD4^+^ T-cells was also observed after stimulation with Group B Streptococcus (GBS), one of the most important pathogens in neonatal sepsis. (4) Expression of the T-cell receptor CD3 and the co-stimulatory molecule CD28 did not differ between neonatal and adult T-cells upon bacterial stimulation.

## Methods

### Patients

The study protocol was approved by the Ethics Committee of the Medical Faculty of Tübingen University (458/2019BO1). All mothers gave written informed consent prior to going into labor. Cord blood was collected from healthy term newborns (≥ 37 + 0 gestational weeks, total *n* = 32) immediately after cesarean section or vaginal delivery and placed in heparin-coated tubes. Collection of cord blood samples was anonymized; thus, no information on sex, exact gestational age, or mode of delivery was available. Children with intra-amniotic infection, defined by the German Society of Gynaecology and Obstetrics as increased maternal inflammatory markers without any other cause (CRP > 10 mg/l or elevation of white blood cell count > 15,000/μL), fetal or maternal tachycardia, maternal fever (≥ 38.0 °C), painful uterus, and foul-smelling amniotic fluid, were excluded. Peripheral blood was collected from trained healthy adult volunteers (total *n* = 28).

### Cell isolation and culture

Mononuclear cells from heparinized cord blood (CBMC) and peripheral blood (PBMC) were isolated by density gradient centrifugation (lymphocyte separation medium, Biochrom, Berlin, Germany). T-cells were separated from PBMC and CBMC after labeling with human CD4^+^ T-cell isolation (Miltenyi Biotec, Bergisch Gladbach, Germany) and separated by magnetic activated cell sorting (MACS) in an autoMACS® separator according to the manufacturer’s protocol (Miltenyi Biotec). Monocytes were separated from PBMC by two consecutive steps of panning (each 2 h) in cell culture flasks.

Cells were then diluted in RPMI 1640 containing 10% heat-inactivated fetal calf serum (FCS, Biochrom, Berlin, Germany), 1% penicillin/streptomycin (Biochrom), and 1% Glutamine (Biochrom) at a concentration of 2 × 10^6^ cells/ml.

For stimulation of PBMC and CBMC with OKT3, cells were seeded to 24 well plates (2 × 10^6^ cells per well) and stimulated with 0.01 µg/ml OKT3 (LEAF purified anti-human CD3 (Clone: OKT3, BioLegend, San Diego, CA, USA).

### Bacterial culture

The culture of Group B streptococci was performed as previously described [25]. Briefly, the GBS strain BSU98 was freshly grown on Columbia agar plates (Sigma, Munich, Germany) supplemented with 5% defibrinated sheep blood, and spectinomycin (150 g/ml; Sigma) for 16 h. Colonies were re-suspended in PBS, and bacterial number was determined spectrometric. Bacteria were inactivated for 30 min at 70 °C. For stimulation of PBMC and CBMC with GBS, cells were seeded in 24 well plates (2 × 10^6^ cells/well) and stimulated with inactivated GBS in a multiplicity of infection (MOI) of 1:50.

A clinical isolate of *E. coli* K1 was grown on agar plates. After 16 h, a single colony was picked and grown in Lennox L broth-medium (Invitrogen, Karlsruhe, Germany) with or without supplements until early logarithmic growth. For stimulation of PBMC and CBMC, cells were seeded to 24 well plates (2 × 10^6^ cells/ml) and stimulated with *E. coli* in a MOI of 1:50.

For inhibition of TRIF, the Pepinh-TRIF inhibitor or the appropriate control peptide (both InvivoGen, France) were added during stimulation with *E. coli*.

### T-cell proliferation assay

For analysis of T-cell proliferation of cord blood and adult T-cells, MACS-isolated T-cells were stained with carboxyfluorescein succinimidyl ester (CFSE, Invitrogen, Heidelberg, Germany) according to the manufacturer’s instructions and seeded into round-bottom 96-well plates (2 × 10^5^ cells in 100 μl RPMI media). Freshly isolated monocytes from a different adult donor were added as co-stimulatory cells in a 1:2 ratio. Cells were stimulated with different concentrations of OKT3 (0.01 μg/ml, 0.1 μg/ml, and 1 μg/ml). After 96 h of incubation, cells were harvested and stained with anti-CD4-APC and anti-CD8-PE. CFSE fluorescence intensity was analyzed by flow cytometry. Data acquisition was performed with a FACSCalibur, and a FACSCantoII flow cytometer and data were analyzed via CellQuest and FlowJo (BD Biosciences).

### Flow cytometry

Antibodies used for extracellular staining of T-cells were purchased from BD Bioscience, Heidelberg, Germany (CD4-FITC, clone SK3, CD8-PerCp, clone SK1, CD28-PE, clone L293 and CTLA-4-PE, clone BNI3, Fixable Viability Stain 510), Miltenyi Biotec (CD3-FITC, clone REA613, CD4-PerCP, clone M-T466, CD69-APC, clone REA824, HLA-DR-PerCP clone REA805), and BioLegend, San Diego, USA (CD3-PE-Cy7, clone HIT3a, CD38-PE, clone S17015F, CD4-Pacific Blue, clone RPA-T4, CD8-APC-Cy7, clone SK1, CD25-BV421 clone M-A251, CD45RO-PerCp, clone UCHL1, CD197-PE, clone G043H7, PD-1-APC clone NAT105, PD-L1-APC clone B7H-1, PD-L2-PE clone 24F.10C12). T-cell subpopulations were defined by staining with CD45RO and CCR7. Naïve T-cells were CD45RO^−^/CCR7^+^, central memory T-cells were CD45RO^+^/CCR7^+^, and effector memory T-cells were CD45RO^+^/CCR7^−^. All antibodies were tested for their specificity by isotype control staining upon introduction to our laboratory. For each antibody, combination compensation with single staining was performed. For the experiments showed here, we used fluorescence minus one (FMO) controls to set thresholds for positive and negative stained cells. Gating strategy for CD4^+^ and CD8^+^ T-cells and strategy to set the threshold for ICM staining is depicted in Supplementary Fig. 2. Data acquisition was performed with a FACSCalibur and FACSCantoII flow cytometer, and data were analyzed via CellQuest and FlowJo (BD Biosciences).

### Statistics

Statistical analyses were done with GraphPad Prism version 9.1.2. Values were tested for Gaussian distribution using D’Agostino and Pearson omnibus normality test. Differences in proliferation assays and differences in the expression of surface ICM (percentages) were analyzed using the Wilcoxon matched-paired signed rank test. Differences in MFI were analyzed using the paired *t* test. A *p* value of < 0.05 was considered statistically significant.

## Results

### Increased proliferation of neonatal T-cells upon stimulation with OKT3

**Fig. 1:**
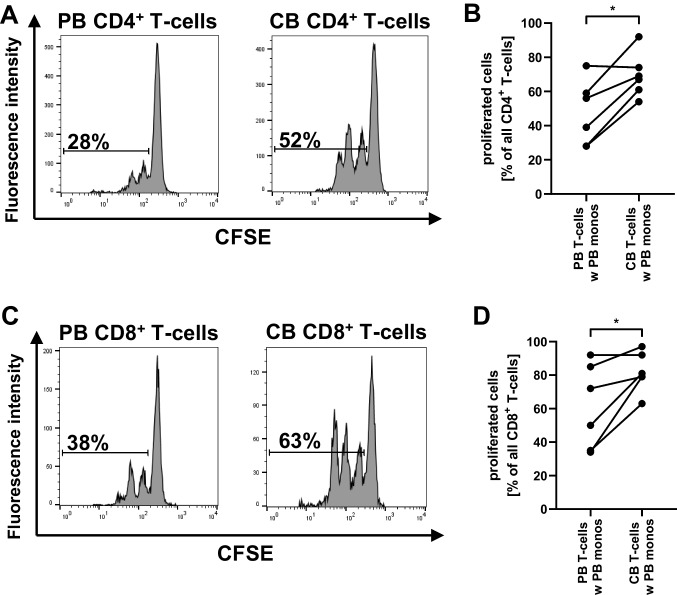
Proliferation of cord blood and adult T-cells upon stimulation with OKT3. T-cells from CBMC or PBMC were enriched by MACS and CFSE-stained. MACS-isolated monocytes from PBMC (different donor) were added in a 1:2 ratio. Co-cultures were stimulated with 0.01 μg/ml OKT3 and after 96 h T-cell proliferation was assessed by flow cytometry. **A**, **C** Histograms show proliferation of CD4^+^ T-cells (**A**) and CD8^+^ T-cells (**C**) isolated from adult blood (left histograms) or cord blood (right histograms). **B**, **D** Scatter plots with connecting lines show proliferation of CD4^+^ (**B**) and CD8^+^ (**D**) T-cells in co-culture with adult monocytes. *n* = 6, *p < 0.05; Wilcoxon matched-pairs signed rank test

First, we asked whether there are differences in the proliferation of adult and neonatal T-cells upon stimulation of the T-cell receptor with OKT3. Therefore, we added monocytes isolated from adult PBMC either to T-cells isolated from CBMC or T-cells isolated from PBMC (different donor) and stimulated cells with OKT3 for 4 days. Here, we found a stronger proliferation in cord blood T-cells than in adult T-cells when both were cultured with adult monocytes (CD4^+^ T-cell proliferation 47.5% ± 18.9% (adult) vs. 69.5% ± 13.0% (cord blood), *n* = 6, p < 0.05, Fig. 1A) and CD8^+^ T-cell proliferation 61.3% ± 25.2% (adult) vs. 81.8% ± 11.9% (cord blood), *n* = 6, *p* < 0.05, Fig. 1B), suggesting that cord blood T-cells get more activated upon T-cell receptor engagement. Increasing the OKT3 concentration did not alter this effect (Supplementary Fig. 1).

### Neonatal T-cells show increased expression of activation markers upon stimulation with OKT3

**Fig. 2:**
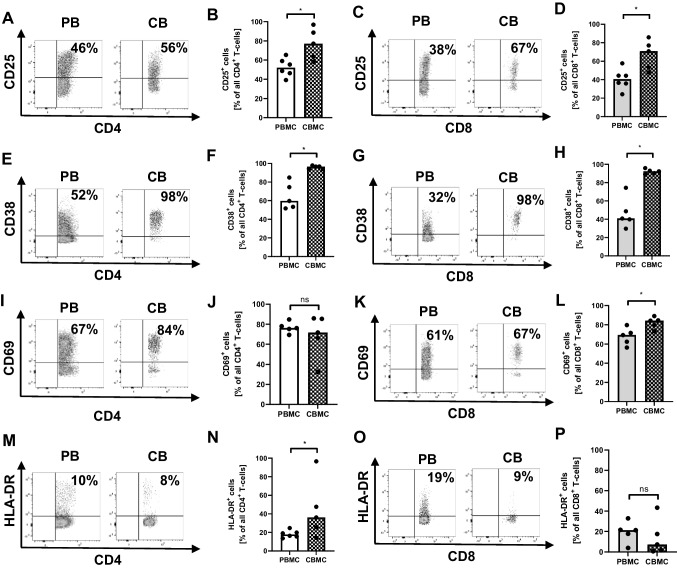
Expression of activation markers on neonatal and adult T-cells upon stimulation with OKT3. Mononuclear cells from cord blood (CBMC) and peripheral blood of healthy adults (PBMC) were isolated and incubated with OKT3 overnight. Expression of surface activation markers on CD4^+^ and CD8^+^ T-cells was determined by flow cytometry. **A**, **C**, **E**, **G**, **I**, **K**, **M**, **O** Representative density plots show percentages of CD25- (**A**, **C**), CD38- (**E**, **G**), CD69- (**I**, **K**) and HLA-DR- (**M**, **O**) expressing CD4^+^ (**A**, **E**, **I**, **M**), and CD8^+^ (**C**, **G**, **K**, **O**) T-cells in adult (left plots) and cord blood (right plots). **B**, **F**, **J**, **N**, **D**, **H**, **L**, **P** Scatter plots with bars show percentages of CD25- (**B**, **D**), CD38- (**F**, **H**), CD69- (**J**, **L**), and HLA-DR-expressing (**N**, **P**) cells on adult (plain bars) and cord blood T-cells (checked bars) after stimulation with OKT3. Bars represent pooled data from 5 independent experiments and each point represents an individual sample. *p < 0.05; ns, not significant; Wilcoxon matched-pairs signed rank test

Next we analyzed expression of T-cell activation markers CD25, CD38, CD69, and HLA-DR on cord blood and adult T-cells. We found that compared to adult cells significantly more cord blood CD4^+^ and CD8^+^ T-cells expressed CD25 (median 77.1% vs. 52.1% for CD4 and 70.8% vs. 40.5% for CD8, *n* = 5, *p* < 0.05, Fig. 2A–D) and CD38 (median 96.6% vs. 59.7% for CD4 and 92.4% vs. 40.8% for CD8, *n* = 5, *p* < 0.05, Fig. 2E–H) upon stimulation with OKT3. More cord blood CD8^+^ T-cells expressed CD69, while on CD4^+^ T-cells, CD69-expression did not differ (median 71.7% vs. 76.1% for CD4 and 84.3% vs. 69.3% for CD8, *n* = 5, *p* < 0.05, Fig. 2I–L). HLA-DR was expressed on more CD4^+^ cord blood T-cells and did not differ between cord blood and adult blood CD8^+^ T-cells (median 36.1% vs. 17.7% for CD4 and 7.2% vs. 21.3% for CD8, *n* = 5, *p* < 0.05, Fig. 2M-P). Supplementary Fig. 2 shows mean fluorescence intensity of CD25, CD38, CD69, and HLA-DR expression on CD4^+^ and CD8^+^ T-cells.

### Neonatal T-cells show decreased expression of ICM upon stimulation with OKT3

**Fig. 3:**
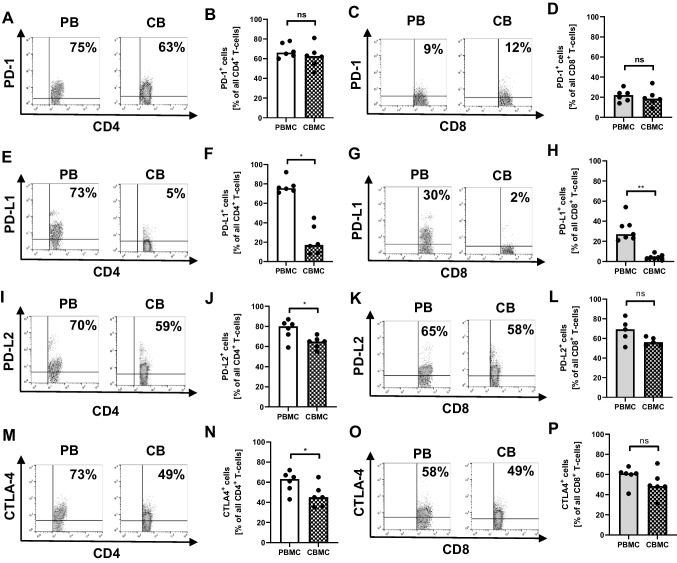
Expression of ICM on neonatal and adult T-cells upon stimulation with OKT3. Mononuclear cells from cord blood (CBMC) and peripheral blood of healthy adults (PBMC) were isolated and incubated with OKT3 overnight. Expression of surface ICM on CD4^+^ and CD8^+^ T-cells was determined by flow cytometry. **A**, **C**, **E**, **G**, **I**, **K**, **M**, **O** Representative density plots show percentages of PD-1- (**A**, **C**), PD-L1- (**E**, **G**), PD-L2- (**I**, **K**), and CTLA-4- (**M**, **O**) expressing CD4^+^ (**A**, **E**, **I**, **M**) and CD8^+^ (**C**, **G**, **K**, **O**) T-cells in adult (left plots) and cord blood (right plots). **B**, **F**, **J**, **N**, **D**, **H**, **L**, **P** Scatter plots with bars show percentages of PD-1- (**B**, **D**), PD-L1- (**F**, **H**), PD-L2- (**J**, **L**), and CTLA-4-expressing (**N**, **P**) cells on adult (plain bars) and cord blood T-cells (checked bars) after stimulation with OKT3. Bars represent pooled data from 6 independent experiments and each point represents an individual sample. *p < 0.05; **p < 0.01; ns, not significant; Wilcoxon matched-pairs signed rank test

We further analyzed percentages of ICM-expressing T-cells (PD-1, PD-L1, PD-L2, and cytotoxic T-lymphocyte-associated protein 4 (CTLA-4)) in adult and cord blood after OKT3 stimulation. Unstimulated CD4^+^ and CD8^+^ T-cells from adult as well as from cord blood did not express PD-1, PD-L1, PD-L2, and CTLA-4 (data not shown). Upon OKT3-stimulation, about 70% of adult CD4^+^ T-cells expressed PD-1 and PD-L1, about 80% expressed PD-L2, and about 60% expressed CTLA-4. Overall, less CD8^+^ T-cells expressed ICM with about 20% of adult CD8^+^ T-cells expressing PD-1, 30% expressing PD-L1, 70% expressing PD-L2, and 60% expressing CTLA-4 (Fig. 3A–P). Significantly less cord blood CD4^+^ and CD8^+^ T-cells expressed PD-L1 compared to adult CD4^+^ and CD8^+^ T-cells (median 17.0% vs. 75.5% for CD4 and 4.0% vs. 27.0% for CD8, *n* = 6–7, *p* < 0.01, Fig. 3E–H), while percentages of PD-L2 and CTLA-4 expressing cells were only lower in cord blood CD4^+^ (median 65.0% vs. 80.0% for PD-L2 and 45.0% vs. 63.0% for CTLA-4, both *n* = 6, *p* < 0.05) but not in cord blood CD8^+^ T-cells (Fig. 3I–P), and percentages of PD-1-expressing cells did not differ significantly in either subpopulations from between cord blood and adult cells (Fig. 3A–D). Supplementary Fig. 3C–J shows mean fluorescence intensity of PD-1, PD-L1, PD-L2, and CTLA-4 expression on CD4^+^ and CD8^+^ T-cells. To exclude that the decreased ICM expression on neonatal T-cells is due to an altered composition of T-cell subpopulations, we analyzed PD-L1 expression on naïve (CD45RO^−^/CCR7^+^), central memory (CD45RO^+^/CCR7^+^), and effector memory (CD45RO^+^/CCR7^−^) T-cell subsets. As expected, we found increased percentages of naïve T-cells and decreased percentages of effector memory CD4^+^ and CD8^+^ T-cells in cord blood samples compared to adult blood (Supplementary Fig. 4A–G). However, decreased expression of PD-L1 on neonatal T-cells affected all T-cell subsets (Supplementary Fig. 4H–P).

### Neonatal CD4^+^ T-cells show decreased upregulation of ICM upon stimulation with GBS

**Fig. 4:**
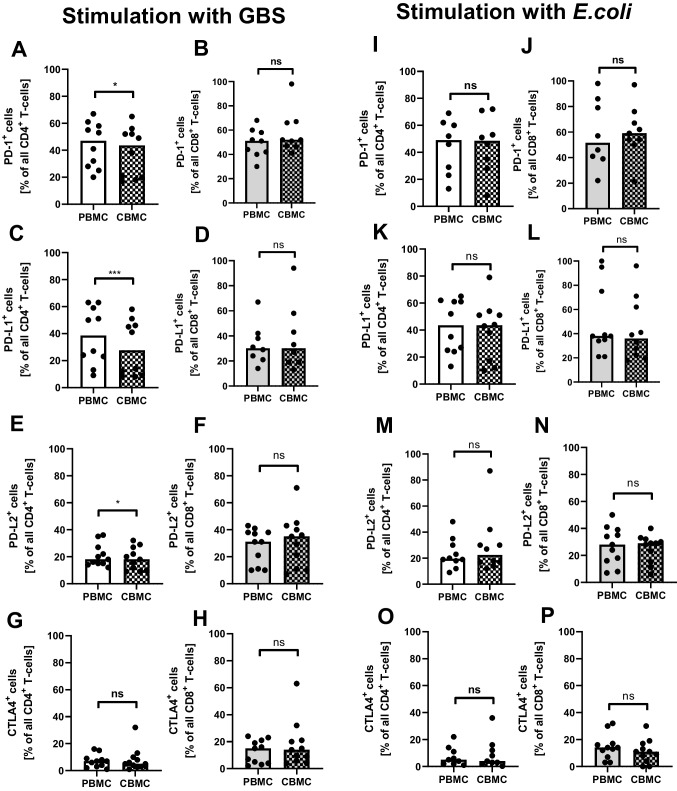
Expression of ICM on neonatal and adult T-cells upon stimulation with GBS and *E. coli.* Mononuclear cells from cord blood (CBMC) and peripheral blood of healthy adults (PBMC) were isolated and incubated with GBS or *E. coli* overnight. Expression of surface ICM on CD4^+^ and CD8^+^ T-cells was determined by flow cytometry. Scatter plots with bars show percentages of PD-1- (**A**, **B**, **I**, **J**), PD-L1- (**C**, **D**, **K**, **L**), PD-L2- (**E**, **F**, **M**, **N**), and CTLA-4- (**G**, **H**, **O**, **P**) expressing CD4^+^ T-cells (**A**–**G**, **I**–**O**) and CD8^+^ T-cells (**B**–**H**, **J**–**P**) from adult blood (plain bars) and cord blood (checked bars) after stimulation with GBS (**A**–**H**) or *E. coli* (**I**–**P**). Bars represent pooled data from 8 to 11 independent experiments and each point represents an individual sample. *p < 0.05; ***p < 0.001; ns, not significant; Mann–Whitney test

We next asked whether the decreased numbers of ICM-expressing cells observed in cord blood T-cells in comparison to adult T-cells upon stimulation with OKT3 also occurs after stimulation with the neonatal pathogens *E. coli* or GBS. Upon stimulation with both, *E. coli* and GBS about 40% of adult CD4^+^ T-cells expressed PD-1 and PD-L1, about 20% PD-L2 and only 10% CTLA-4 (Fig. 4A–P and Supplementary Figs. 5–6). Interestingly, upon stimulation with *E. coli*, percentages of ICM-expressing cells did not differ between cord blood and adult CD4^+^ T-cells for all ICM analyzed, while stimulation with GBS again led to a significantly lower percentages of PD-1- (median 43.5% vs. 47.0%, *n* = 10, *p* < 0.05, Fig. 4A and Supplementary Fig. 5A), PD-L1- (median 27.5% vs. 38.5%, *n* = 10, *p* < 0.01, Fig. 4C and Supplementary Fig. 5B), and PD-L2-expressing cells (median 16.0% vs. 18.0%, *n* = 11, *p* < 0.05, Fig. 4E and Supplementary Fig. 5C) in cord blood CD4^+^ T-cells in comparison to adult CD4^+^ T-cells. In CD8^+^ T-cells, we found no differences in percentages of PD-1-, PD-L1-, PD-L2-, and CTLA-4-expressing cells upon stimulation with *E. coli* or GBS between cord blood and adult blood (Fig. 4B, D, F, H, J, L, N, P and Supplementary Figs. 5–6). Corresponding to this, we found increased proliferation of cord blood CD4^+^ T-cells after stimulation with GBS and OKT3, but not after stimulation wit E. coli and OKT3 (Supplementary Fig. 7).

### Expression of T-cell receptor CD3 and co-stimulatory CD28 does not change between neonatal and adult T-cells upon stimulation with E. coli or GBS

**Fig. 5:**
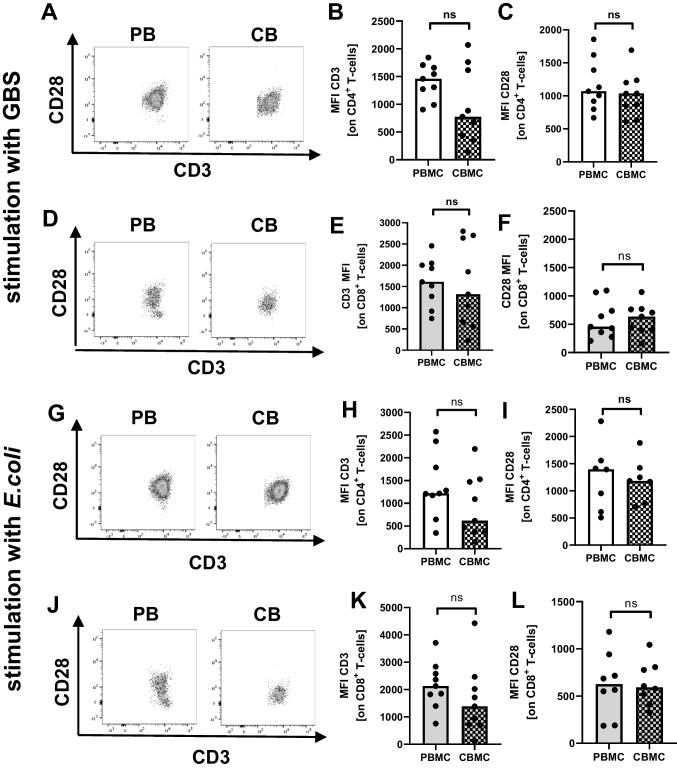
Expression of CD3 and CD28 on neonatal and adult T-cells upon stimulation with *E. coli* or GBS. Mononuclear cells from cord blood (CBMC) and peripheral blood of healthy adults (PBMC) were isolated and incubated with GBS or *E. coli* overnight. Expression of the T-cell receptor CD3 and the co-stimulatory molecule CD28 on CD4^+^ and CD8^+^ T-cells was determined by flow cytometry. **A**, **D**, **G**, **J** Representative density plots show expression of CD3 and CD28 on CD4^+^ (**A**, **D**) and CD8^+^ (**G**, **J**) T-cells in adult (left plots) and cord blood (right plots). **B**, **C**, **E**, **F**, **H**, **I**, **K**, **L** Scatter plots with bars show the mean fluorescent intensity (MFI) for expression of CD3 (**B**, **E**, **H**, **K**) and CD28 (**C**, **F**, **I**, **L**) on CD4^+^ T-cells (**B**, **C**, **H**, **I**), and CD8^+^ T-cells (**E**, **F**, **K**, **L**) after stimulation with GBS (**B**, **C**, **E**, **F**) or *E. coli* (**H**, **I**, **K**, **L**). Bars represent pooled data from 7 to 9 independent experiments, and each point represents an individual sample. ns, not significant; Mann–Whitney test

Lastly, we analyzed if the expression of the T-cell receptor (CD3) and the co-stimulatory molecule CD28 upon stimulation with *E. coli* or GBS also differ between cord blood and adult T-cells. For both, stimulation with *E. coli* and GBS and expression of CD3 and CD28 did not differ between cord and adult blood CD4 + and CD8 + T-cells (Fig. 5A–L). In cord blood CD4^+^ T-cells, there was even a tendency for a decreased expression of CD3 upon stimulation wit *E. coli* or GBS.

## Discussion

It is well known that term and especially preterm infants are predisposed to bacterial infections such as sepsis. Moreover, the termination of inflammation seems to be disturbed leading to sustained inflammation [1, 26]. This contributes to the development of post-inflammatory diseases such as bronchopulmonary dysplasia (BPD) and periventricular leukomalacia (PVL), thereby influencing short- and long-term outcome [23].

In the present study, we found that in presence of adult monocytes (1) neonatal T-cells exhibited an increased proliferative capacity and an increased expression of T-cell activation markers in comparison to adult T-cells upon stimulation with OKT3. (2) This was accompanied by a decreased expression of ICM on neonatal T-cells, especially on CD4^+^ T-cells. (3) Also, upon stimulation with GBS, but not with *E. coli*, expression of ICM, especially that of PD-L1, was decreased on neonatal compared to adult CD4^+^ T-cells, while (4) expression of the T-cell receptor CD3 and the co-stimulatory molecule CD28 did not differ between neonatal and adult T-cells.

Our finding of increased proliferation of neonatal T-cells in comparison to adult T-cells is in line with previous reports also showing an increased proliferative response of cord blood T-cells stimulated by adult APC in comparison to adult T-cells [27–29], while cytokine production and cytotoxic activity of neonatal T-cells seem to be diminished in comparison to adult T-cells [27, 28, 30–32] potentially contributing to decreased graft versus host disease after transplantation of umbilical cord blood [33]. When total mononuclear cells were analyzed, cord blood T-cells were shown to proliferate to a lesser extent in comparison to adult T-cells, potentially due to a decreased co-stimulatory activity of cord blood monocytes [29, 34] and/or the presence of immune-suppressive cells among cord-blood mononuclear cells [35]. Interestingly, we and others recently showed that after bacterial stimulation proliferation of adult T-cells is strongly suppressed, while proliferation of neonatal T-cells is unaffected or even increased [11, 36], suggesting that neonatal T-cells have a high proliferative potential that is normally kept in check but may become relevant during infection/inflammation.

In addition to the increased proliferation after OKT3 stimulation, we observed increased expression of activation markers CD25, CD38, CD69, and HLA-DR on neonatal compared to adult T-cells. In particular, a greater proportion of neonatal T-cells expressed CD25 and CD38. CD25 is part of the receptor for interleukin-2 (IL-2R) which is one of the cytokines inducing T-cell-proliferation, and increased expression of CD25 on neonatal T-cells may reflect increased susceptibility to IL-2-induced proliferation. The increased expression of CD38 on neonatal T-cells after OKT3 stimulation that accompanied their increased proliferative capacity in comparison to adult T-cells is in contrast to observations from Sandoval-Montes et al. who showed that T-cells expressing high levels of CD38 had a reduced proliferative capacity but an increased potential to produce pro-inflammatory cytokines [37]. Despite this contradiction, our results of increased proliferation and increased expression of activation markers on neonatal T cells in comparison to adult T-cells, suggest that neonatal T-cells have an enhanced ability to response to stimulation.

After stimulation with OKT3, especially neonatal CD4^+^ T-cells showed a decreased expression of ICM compared to adult T-cells. This was confirmed, albeit to a lesser extent, after stimulation with GBS but not with *E. coli*. Co-stimulatory and co-inhibitory molecules elementarily determine the functional outcome of T-cell receptor signaling [38]. ICM can induce and maintain immunologic self-tolerance and on the other hand control the duration and extent of immune responses, thereby minimizing tissue damage [39, 40]. A disturbed balance between co-stimulation and co-inhibition can lead to pathological conditions such as tumors and autoimmune disorders [41, 42]. The role of ICM in neonatal T-cell response, in particular during and after neonatal sepsis, is poorly understood. Walk et al. investigated expression of seven ICM on cord blood cells with a focus on innate immune cells. They found an increased expression of LAIR-1, CD31, and CD200 on neonatal T-cells in comparison to adult T-cells hypothesizing that this may play a role in dampening the adaptive immune response in utero [43]. Corresponding to our results, Miller et al. found a decreased mRNA and protein expression of CTLA-4 in cord blood T-cells in comparison to adult T-cells [44]. To the best of our knowledge, no descriptive data for PD-L1 and PD-1 in neonatal T-cells exist. Zasada et al. showed that in all monocyte subsets PD-1 receptors were present on the 5th postnatal day, but neonatal T-cells were not investigated [45]. Data of the same study showed that monocytes from VLBW infants with late-onset-sepsis expressed a lower percentage of PD-1 receptors, but the PD-1 expression was elevated. Young et al. showed that a lack of PD-1 led to improved survival during neonatal polymicrobial sepsis [46]; however, the cell type responsible for this is unknown. Our findings of a diminished expression of ICM on neonatal in comparison to adult T-cells may suggest that neonatal T-cells are less able to enter an anergic state and terminate the inflammatory response, which could then contribute to the development of post-inflammatory complications such as BPD or PVL.

If the decreased expression of PD-L1 and to a lesser extent PD-L2 and CTLA-4 we observed on neonatal T-cells in comparison to adult T-cells after stimulation is the reason for their increased proliferative capacity remains unclear. While signaling via PD-1 in T-cells has been studied in detail (reviewed for example in [47]), the signaling cascade downstream of PD-L1 in T-cells is much less well understood. It has been shown that the intracytoplasmic tail of PD-L1 can trigger a signal cascade that make cancer cells resistant to interferon-mediated cytotoxicity through a STAT3/caspase-7-dependent pathway [48] and that in CD4^+^ T-cells binding to PD-L1 induced STAT3-dependent “back signaling” that prevented T-cell activation and polarization [49]. Recently, Fanelli et al. showed that cross-linking of PD-L1 on CD4^+^ T-cells, induced their conversion into Tregs via the mitogen-activated protein kinase (MAPK) pathway [50], which could be a potential backward mechanism by which PD-L1 expression on T-cells leads to reduced T-cell proliferation. Another mechanism may be a T-T-cell interaction between PD-L1 or PD-L2 expressed on one T-cell and PD-1 expressed on another T-cell leading to inhibition of activation of the PD-1 expressing cell by binding to the PD-L1/PD-L2 expressing cell. More work is needed to figure out whether reduced PD-L1/PD-L2 expression on neonatal T-cells is actually causative for their increased proliferative capacity and if so, what the underlying mechanisms are.

In some samples, we observed two populations of PD-L1-expressing cells of which one expressed PD-L1 very high. We do not have a clear explanation for this. As mentioned before, Fanelli et al. recently showed that PD-L1 ligation induced a highly suppressive phenotype in CD4^+^ T-cells. This was particularly the case in memory T-cells [50]. Interestingly, when we analyzed T-cell subpopulations (see Supplementary Fig. 4), we saw especially high PD-L1 expressions also on central memory T-cells, and it can be speculated that PD-L1 high-expressing CD4^+^ T-cells may represent cells with particularly high potential for adopting regulatory capabilities.

Interestingly, we only found differences in expression of ICM between neonatal and adult T-cells after stimulation with GBS but not after stimulation with *E. coli*. Recognition of GBS by host immune cells mainly involves toll like receptor 2 (TLR2) and myeloid differentiation factor 88 (MyD88) [51], while *E. coli* is recognized by TLR4 and binding activates two alternative adaptor proteins, MyD88a and TIR domain-containing adapter-inducing interferon β (TRIF) [52]. For monocytes, it was shown that TLR-4 expression was reduced in preterm and newborn infants in comparison to adults, while TLR2 expression did not differ between age groups [53, 54]. In addition, it has been shown that in monocytes from adults genes involved in MyD88 signaling were enriched after stimulation with LPS, while in cord blood monocytes, TRIF-signaling pathways were overrepresented pointing to a differential usage of defined signaling pathways in adult and neonatal monocyte downstream of TLR4 [55]. These differences in signaling between neonatal and adult monocytes could be attributed to the presence of high levels of S100 alarmins in the newborn, which lead to epigenetic modification of MyD88-dependent proinflammatory genes in the resting state but prevent activation on stimulation [55]. If these differences in signal transduction are also present in T-cells, this could be an explanation that the lack of PD-L1 upregulation observed in neonatal T-cells upon stimulation with GBS, which is mainly transduced by MyD88, which is weak after bacterial stimulation in neonates, does not occur upon stimulation with *E. coli*, which is transduced via both MyD88 and TRIF, which is well developed in neonates. However, we did not observe in our experiments that upregulation of PD-L1 expression in neonatal T-cells upon *E. coli* stimulation could be reversed by inhibition of TRIF (Supplementary Fig. 8). Further studies are needed to elucidate the underlying mechanisms for the decreased expression of ICM on neonatal T-cells upon activation, especially after GBS challenge. Interestingly, one study showed an increased risk of PVL in infants after GBS sepsis in comparison to other pathogens [56]; however, more evidence that GBS is more likely to cause post-inflammatory diseases is lacking. Further studies are needed to evaluate the impact of certain pathogens on pathogenesis of post-inflammatory diseases and the role of ICM in this context.

In vitro studies with adult cells showed a reduced lymphocyte apoptosis and recovery of effector immune cells after blocking PD-1 and PD-L1 in adult septic patients [57–60]. In a mouse model of intracerebral hemorrhage, PD-L1 reduced the number of CD4^+^ T-cells, decreased cell death, and enhanced the blood–brain barrier integrity [61–63]. These data emphasize our hypothesis that the reduced expression of PD-L1 on CD4^+^ T-cells may contribute to the sustained inflammation of the lung and brain in neonates, but further investigations are needed.

Lastly, we found no changes in the expression of T-cell receptor (TCR) and co-stimulatory molecule CD28 upon stimulation with *E. coli* or GBS in neonates compared to adults, suggesting that TCR signaling and co-stimulation are not impaired in neonates. This is in line with previous studies also showing similar expression of CD3 and CD28 after stimulation with PMA in cord blood and adult T-cells [29, 64, 65]. Furthermore, activation of neonatal T-cells via CD3/CD28 determined by CD25 expression was found to be similar to that of adult T-cells, while cytokine profiles differ significantly. These and our results suggest that not TCR-signaling per se is impaired in neonatal T-cells but rather the downstream mechanisms seem to be regulated differently.

In conclusion, we show that neonatal T-cells have increased proliferative capacity upon stimulation with OKT3 in comparison to adult T-cells accompanied by a decreased expression of ICM. Decreased expression of ICM, especially PD-L1, was also observed after stimulation with GBS but not with *E. coli*. Decreased expression of ICM upon T-cell activation in neonatal cells may be a reason for the increased risk of term and preterm neonates to develop post-inflammatory diseases. More studies are needed to figure out if targeting immune checkpoint molecules in neonates may be a strategy to improve neonatal outcome.

## Supplementary Information

Below is the link to the electronic supplementary material.

Supplementary file1 (PPTX 1105 KB)

## Abbreviations

BPD: Bronchopulmonary dysplasia
CBMC: Cord blood mononuclear cell
CFSE: Carboxyfluorescein succinimidyl ester
CRP: C-reactive protein
CTLA-4: Cytotoxic T-lymphocyte-associated protein 4
*E. coli*: *Escherichia coli*
FCS: Fetal calf serum
FMO: Fluorescence minus one
GBS: Group B Streptococcus
ICM: Immune-checkpoint molecule
MACS: Magnetic-activated cell sorting
MFI: Mean fluorescent intensity
MNC: Mononuclear cells
PBMC: Peripheral blood mononuclear cell
PBS: Phosphate-buffered saline
PD-1: Programmed cell death protein 1
PD-L1: Programmed cell death ligand 1
PD-L2: Programmed cell death ligand 2
PVL: Periventricular leukomalacia
TCR: T-cell receptor
VLBWI: Very low birth weight infant
