# Supplementary material for: Expression of immune checkpoint molecules on adult and neonatal T-cells
Source: Immunol Res. 2022 Nov 23;71(2):185–96. doi: 10.1007/s12026-022-09340-6 (PMC10060332; doi:10.1007/s12026-022-09340-6)
Supplement: Supplementary file 1 — Supplementary file1 (PPTX 1105 KB) [file 12026_2022_9340_MOESM1_ESM.pptx]

## Slide 1
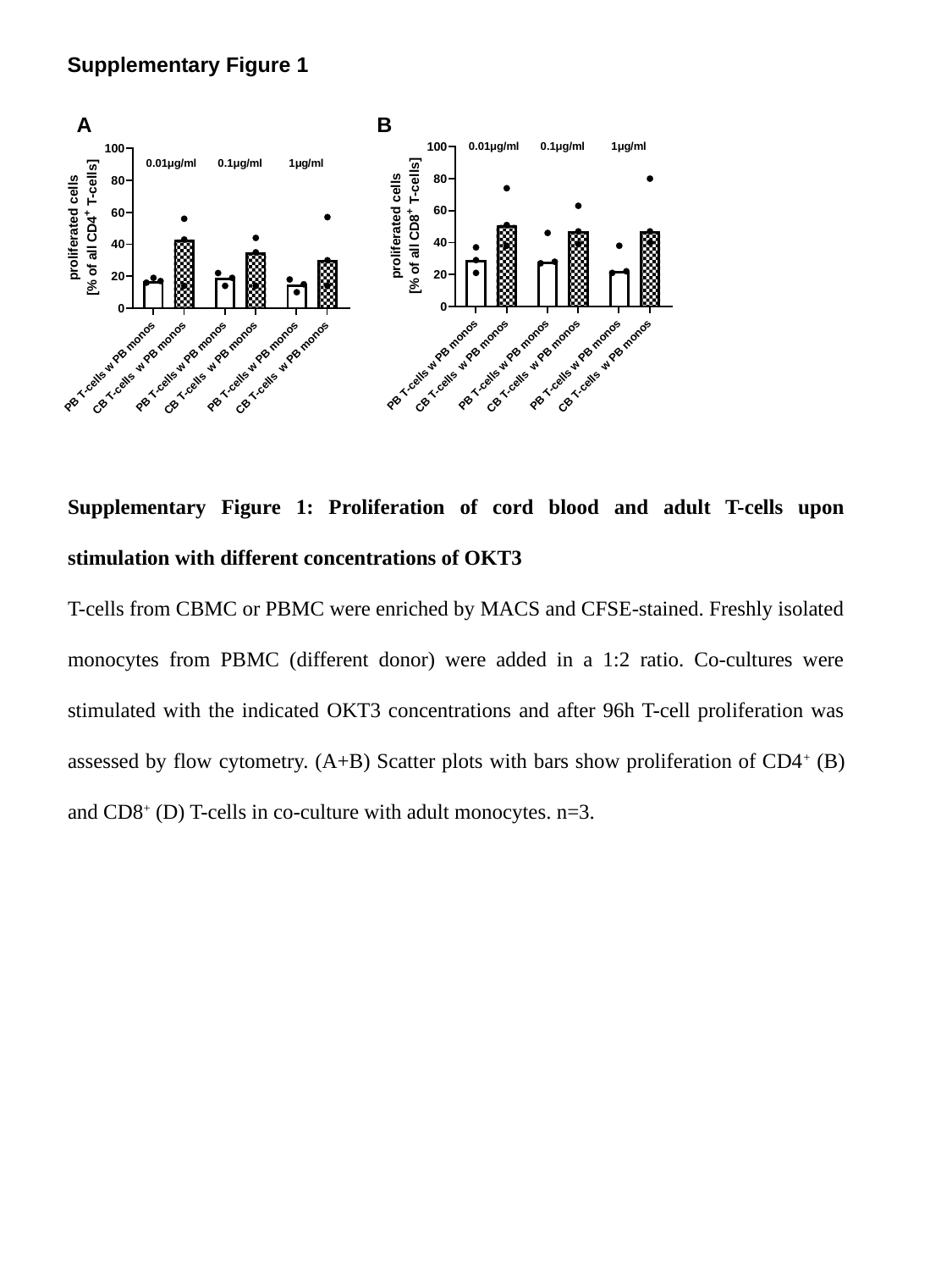

Supplementary Figure 1
A
B
Supplementary Figure 1: Proliferation of cord blood and adult T-cells upon stimulation with different concentrations of OKT3
T-cells from CBMC or PBMC were enriched by MACS and CFSE-stained. Freshly isolated monocytes from PBMC (different donor) were added in a 1:2 ratio. Co-cultures were stimulated with the indicated OKT3 concentrations and after 96h T-cell proliferation was assessed by flow cytometry. (A+B) Scatter plots with bars show proliferation of CD4+ (B) and CD8+ (D) T-cells in co-culture with adult monocytes. n=3.

## Slide 2
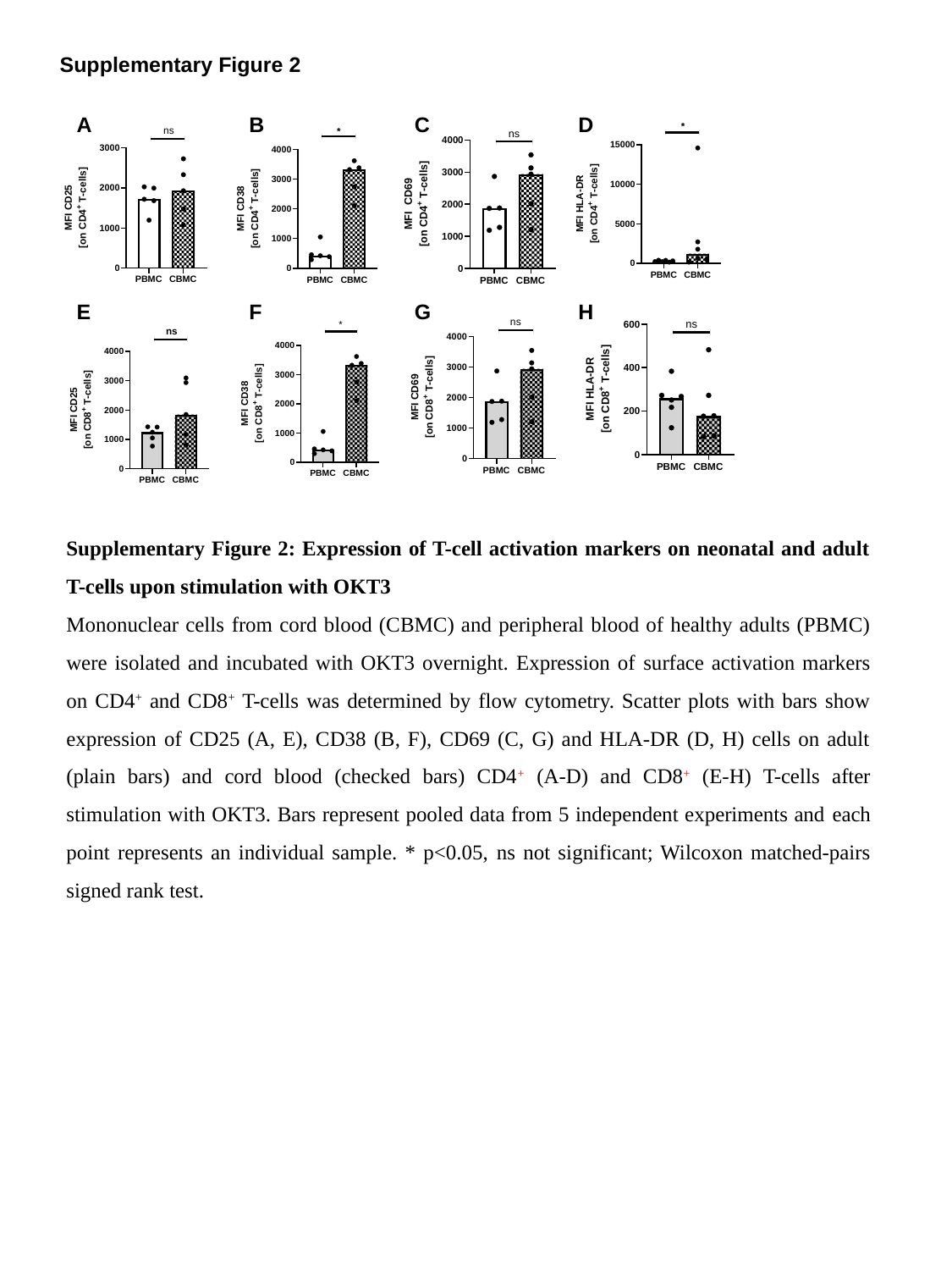

Supplementary Figure 2
A
B
C
D
E
F
G
H
Supplementary Figure 2: Expression of T-cell activation markers on neonatal and adult T-cells upon stimulation with OKT3
Mononuclear cells from cord blood (CBMC) and peripheral blood of healthy adults (PBMC) were isolated and incubated with OKT3 overnight. Expression of surface activation markers on CD4+ and CD8+ T-cells was determined by flow cytometry. Scatter plots with bars show expression of CD25 (A, E), CD38 (B, F), CD69 (C, G) and HLA-DR (D, H) cells on adult (plain bars) and cord blood (checked bars) CD4+ (A-D) and CD8+ (E-H) T-cells after stimulation with OKT3. Bars represent pooled data from 5 independent experiments and each point represents an individual sample. * p<0.05, ns not significant; Wilcoxon matched-pairs signed rank test.

## Slide 3
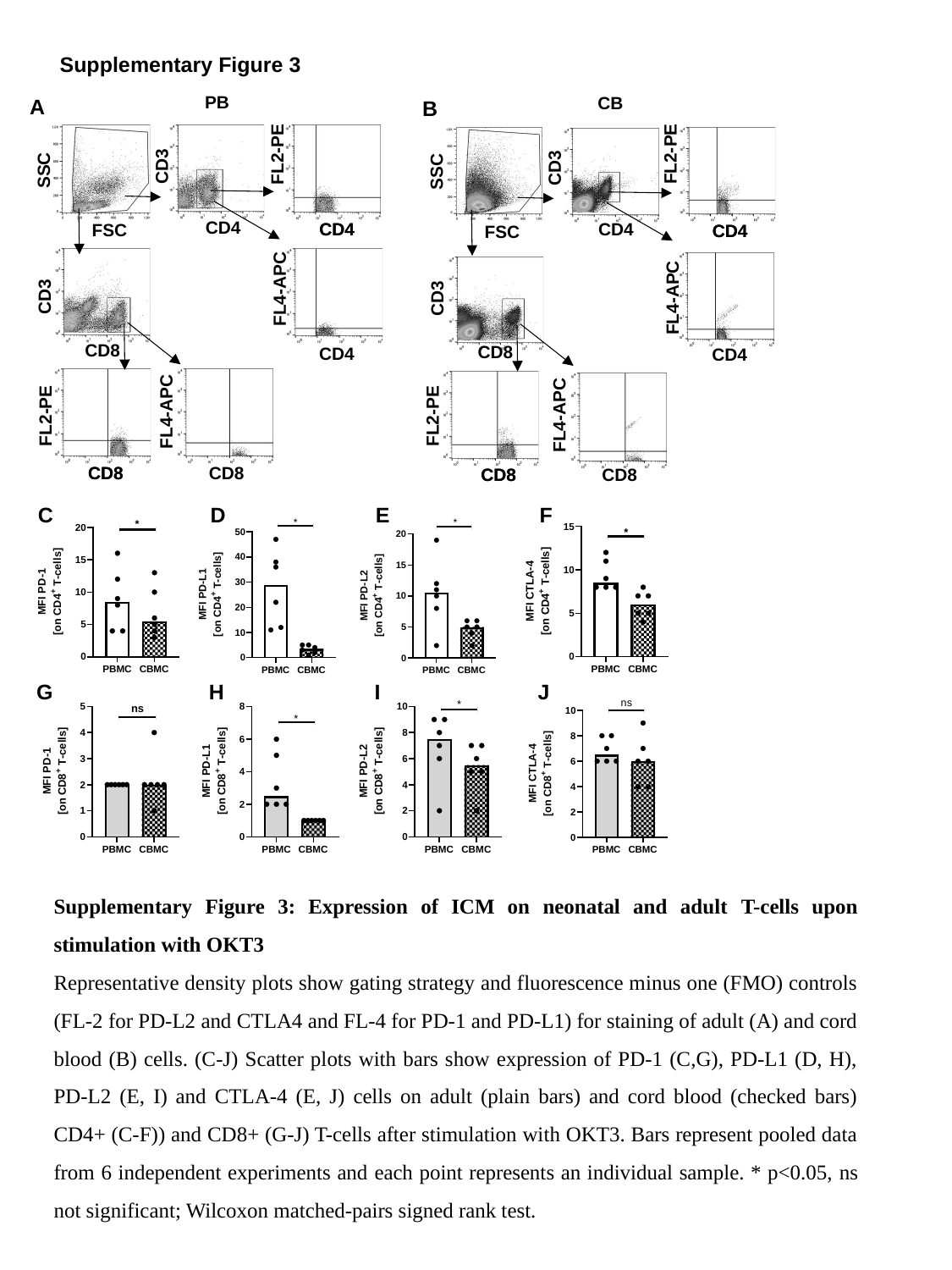

Supplementary Figure 3
PB
CB
A
B
FL2-PE
CD3
FL2-PE
CD3
SSC
SSC
CD4
CD4
CD4
CD4
FSC
CD4
CD4
FSC
CD3
CD3
FL4-APC
FL4-APC
CD8
CD8
CD4
CD4
FL2-PE
FL2-PE
FL4-APC
FL4-APC
CD8
CD8
CD8
CD8
CD8
CD8
C
D
E
F
G
H
I
J
Supplementary Figure 3: Expression of ICM on neonatal and adult T-cells upon stimulation with OKT3
Representative density plots show gating strategy and fluorescence minus one (FMO) controls (FL-2 for PD-L2 and CTLA4 and FL-4 for PD-1 and PD-L1) for staining of adult (A) and cord blood (B) cells. (C-J) Scatter plots with bars show expression of PD-1 (C,G), PD-L1 (D, H), PD-L2 (E, I) and CTLA-4 (E, J) cells on adult (plain bars) and cord blood (checked bars) CD4+ (C-F)) and CD8+ (G-J) T-cells after stimulation with OKT3. Bars represent pooled data from 6 independent experiments and each point represents an individual sample. * p<0.05, ns not significant; Wilcoxon matched-pairs signed rank test.

## Slide 4
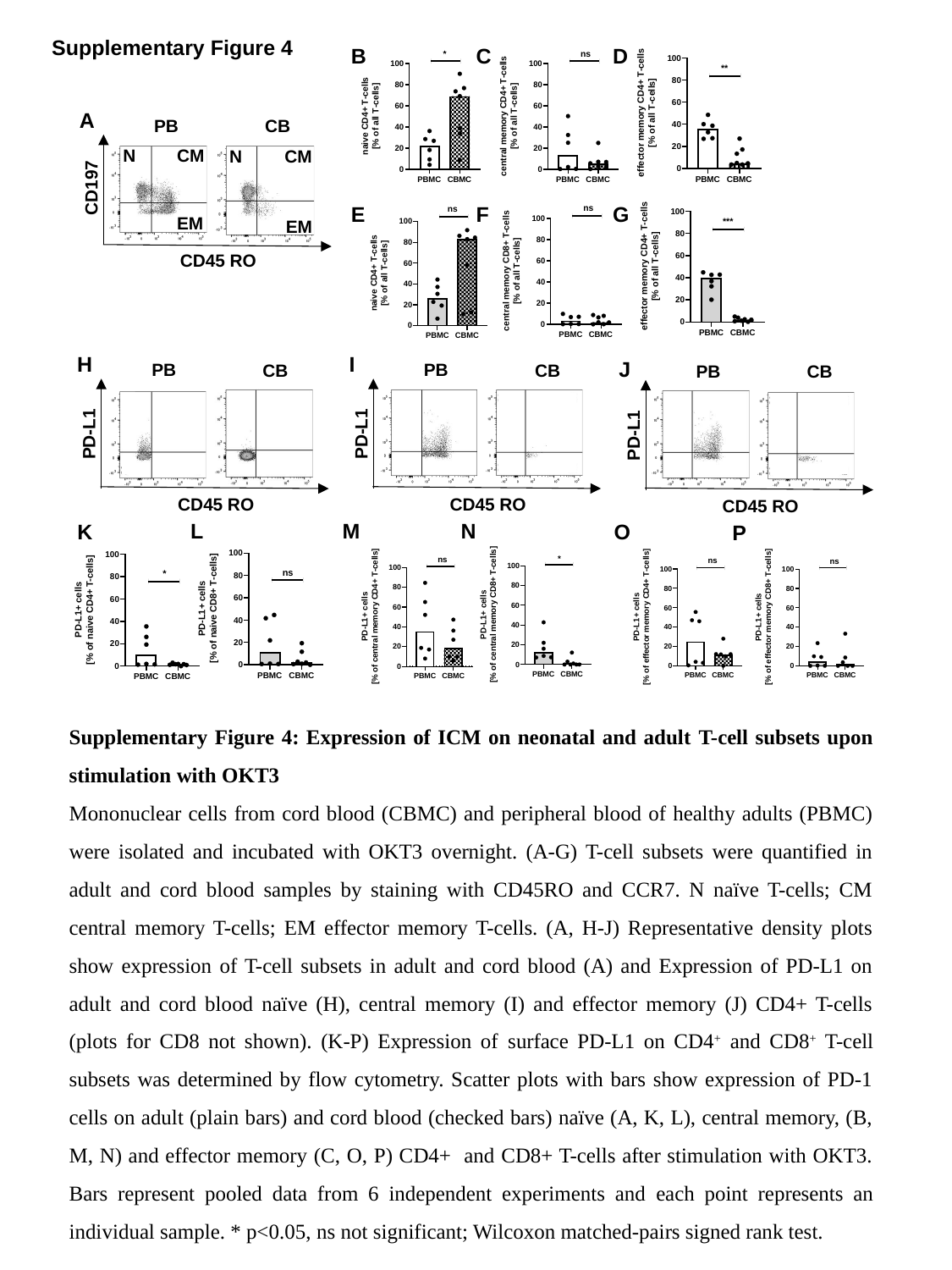

Supplementary Figure 4
C
D
B
A
PB
CB
CM
N
CM
N
CD197
F
G
E
EM
EM
CD45 RO
I
H
J
PB
PB
CB
CB
PB
CB
PD-L1
PD-L1
PD-L1
CD45 RO
CD45 RO
CD45 RO
N
M
L
O
K
P
Supplementary Figure 4: Expression of ICM on neonatal and adult T-cell subsets upon stimulation with OKT3
Mononuclear cells from cord blood (CBMC) and peripheral blood of healthy adults (PBMC) were isolated and incubated with OKT3 overnight. (A-G) T-cell subsets were quantified in adult and cord blood samples by staining with CD45RO and CCR7. N naïve T-cells; CM central memory T-cells; EM effector memory T-cells. (A, H-J) Representative density plots show expression of T-cell subsets in adult and cord blood (A) and Expression of PD-L1 on adult and cord blood naïve (H), central memory (I) and effector memory (J) CD4+ T-cells (plots for CD8 not shown). (K-P) Expression of surface PD-L1 on CD4+ and CD8+ T-cell subsets was determined by flow cytometry. Scatter plots with bars show expression of PD-1 cells on adult (plain bars) and cord blood (checked bars) naïve (A, K, L), central memory, (B, M, N) and effector memory (C, O, P) CD4+ and CD8+ T-cells after stimulation with OKT3. Bars represent pooled data from 6 independent experiments and each point represents an individual sample. * p<0.05, ns not significant; Wilcoxon matched-pairs signed rank test.

## Slide 5
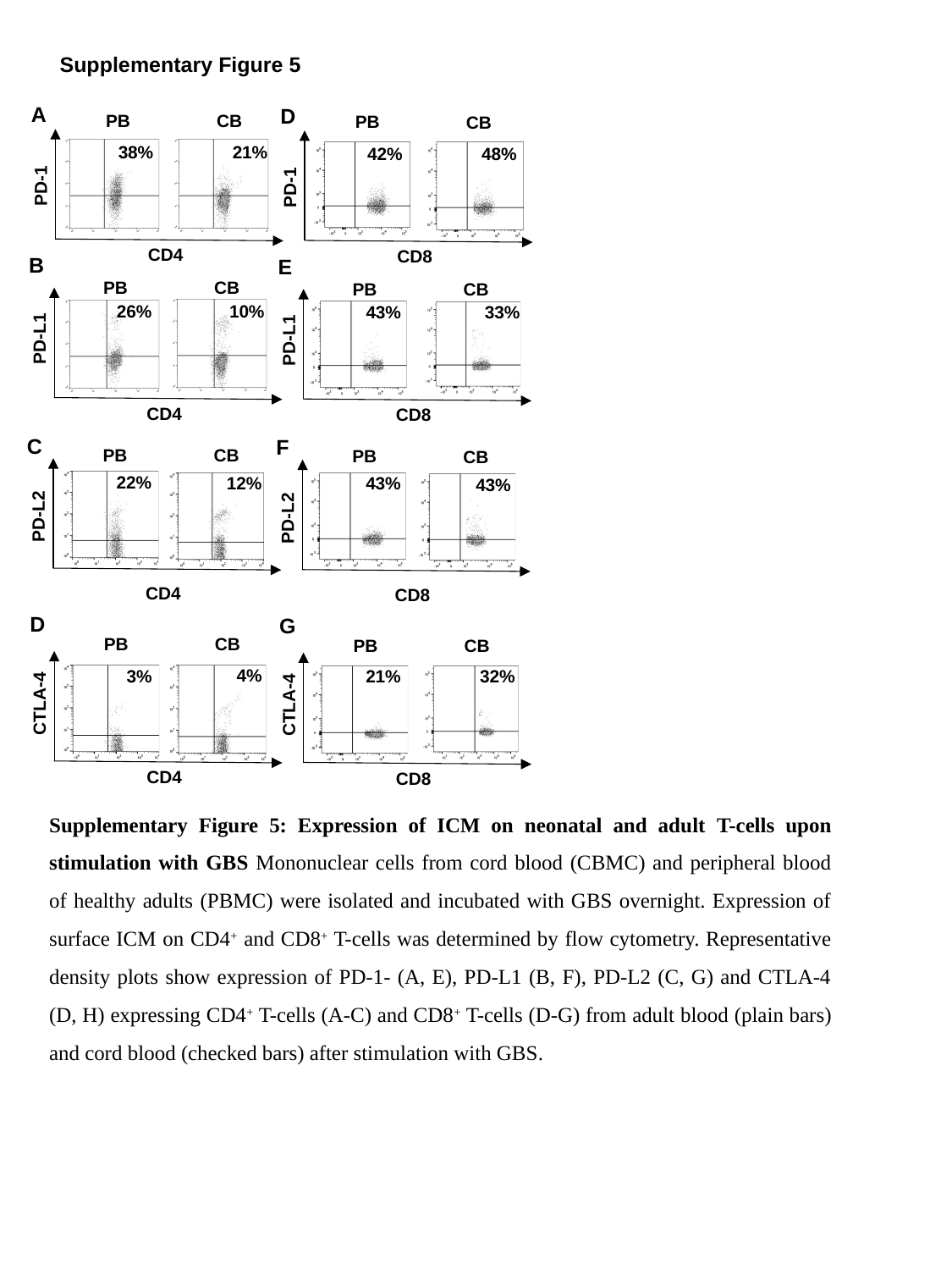

Supplementary Figure 5
A
D
PB
CB
PB
CB
21%
38%
48%
42%
PD-1
PD-1
CD4
CD8
B
E
PB
CB
PB
CB
26%
10%
43%
33%
PD-L1
PD-L1
CD4
CD8
C
F
PB
CB
PB
CB
22%
12%
43%
43%
PD-L2
PD-L2
CD4
CD8
D
G
PB
CB
PB
CB
4%
3%
21%
32%
CTLA-4
CTLA-4
CD4
CD8
Supplementary Figure 5: Expression of ICM on neonatal and adult T-cells upon stimulation with GBS Mononuclear cells from cord blood (CBMC) and peripheral blood of healthy adults (PBMC) were isolated and incubated with GBS overnight. Expression of surface ICM on CD4+ and CD8+ T-cells was determined by flow cytometry. Representative density plots show expression of PD-1- (A, E), PD-L1 (B, F), PD-L2 (C, G) and CTLA-4 (D, H) expressing CD4+ T-cells (A-C) and CD8+ T-cells (D-G) from adult blood (plain bars) and cord blood (checked bars) after stimulation with GBS.

## Slide 6
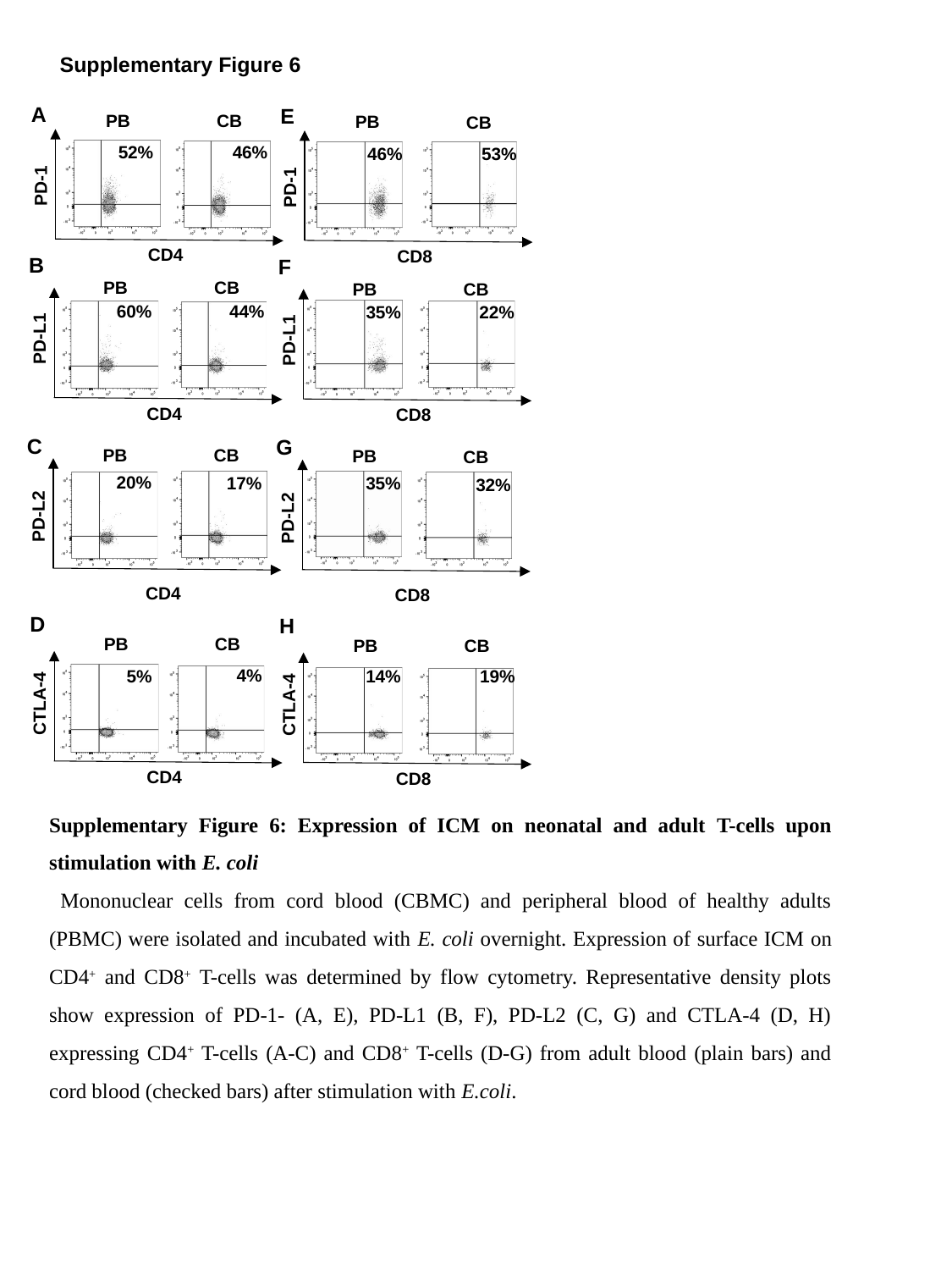

Supplementary Figure 6
A
E
PB
CB
PB
CB
46%
52%
53%
46%
PD-1
PD-1
CD4
CD8
B
F
PB
CB
PB
CB
60%
44%
35%
22%
PD-L1
PD-L1
CD4
CD8
C
G
PB
CB
PB
CB
20%
17%
35%
32%
PD-L2
PD-L2
CD4
CD8
D
H
PB
CB
PB
CB
4%
5%
14%
19%
CTLA-4
CTLA-4
CD4
CD8
Supplementary Figure 6: Expression of ICM on neonatal and adult T-cells upon stimulation with E. coli
 Mononuclear cells from cord blood (CBMC) and peripheral blood of healthy adults (PBMC) were isolated and incubated with E. coli overnight. Expression of surface ICM on CD4+ and CD8+ T-cells was determined by flow cytometry. Representative density plots show expression of PD-1- (A, E), PD-L1 (B, F), PD-L2 (C, G) and CTLA-4 (D, H) expressing CD4+ T-cells (A-C) and CD8+ T-cells (D-G) from adult blood (plain bars) and cord blood (checked bars) after stimulation with E.coli.

## Slide 7
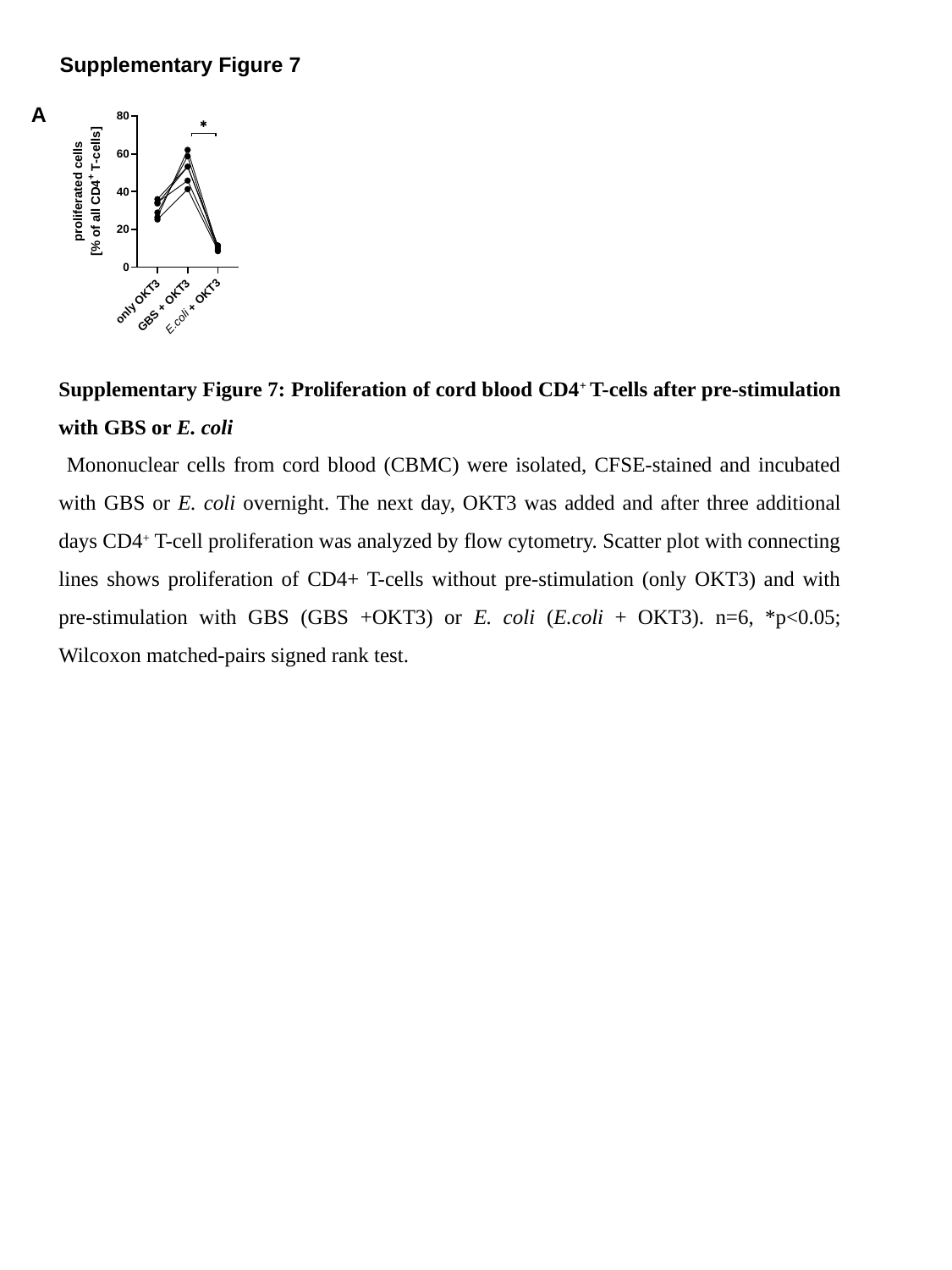

Supplementary Figure 7
A
Supplementary Figure 7: Proliferation of cord blood CD4+ T-cells after pre-stimulation with GBS or E. coli
 Mononuclear cells from cord blood (CBMC) were isolated, CFSE-stained and incubated with GBS or E. coli overnight. The next day, OKT3 was added and after three additional days CD4+ T-cell proliferation was analyzed by flow cytometry. Scatter plot with connecting lines shows proliferation of CD4+ T-cells without pre-stimulation (only OKT3) and with pre-stimulation with GBS (GBS +OKT3) or E. coli (E.coli + OKT3). n=6, *p<0.05; Wilcoxon matched-pairs signed rank test.

## Slide 8
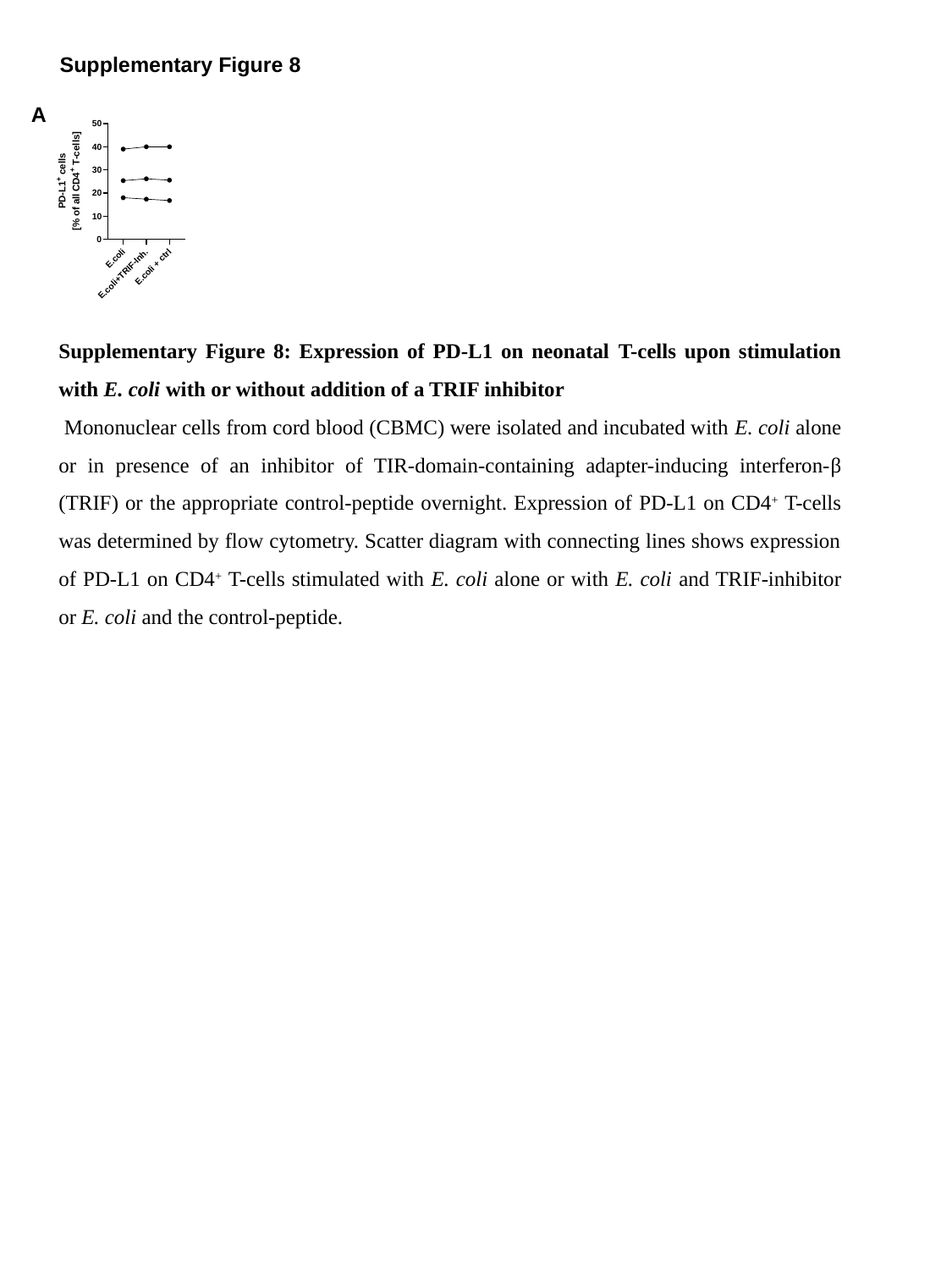

Supplementary Figure 8
A
Supplementary Figure 8: Expression of PD-L1 on neonatal T-cells upon stimulation with E. coli with or without addition of a TRIF inhibitor
 Mononuclear cells from cord blood (CBMC) were isolated and incubated with E. coli alone or in presence of an inhibitor of TIR-domain-containing adapter-inducing interferon-β (TRIF) or the appropriate control-peptide overnight. Expression of PD-L1 on CD4+ T-cells was determined by flow cytometry. Scatter diagram with connecting lines shows expression of PD-L1 on CD4+ T-cells stimulated with E. coli alone or with E. coli and TRIF-inhibitor or E. coli and the control-peptide.
